# Sperm in peritoneal fluid from a man with ascites: a case report

**DOI:** 10.1186/1757-1626-2-192

**Published:** 2009-11-12

**Authors:** Horatiu Olteanu, Alexandra Harrington, Steven H Kroft

**Affiliations:** 1 Department of Pathology, Medical College of Wisconsin, 8701 Watertown Plank Road, Milwaukee, WI, 53226, USA

## Abstract

**Introduction:**

The finding of sperm in body fluids such as peritoneal fluid is unusual, and has been mostly described in female patients.

**Case presentation:**

We are reporting the case of a 52-year-old man who presented with complaints of increased abdominal girth, weight gain and epigastric pain. He was subsequently found to have spontaneous bacterial peritonitis and sperm in the peritoneal fluid. We describe the laboratory findings and clinical course in this patient.

**Conclusion:**

To our knowledge, this is the first report of sperm in peritoneal fluid in a male patient.

## Case presentation

A 52-year-old Indian man with a history of cirrhosis, alcoholic liver disease, esophageal varices, hemolytic anemia, and type 2 diabetes mellitus presented with a 2-week history of increased abdominal girth, 15 pounds of weight gain and epigastric pain. He also complained of decreased energy and shortness of breath with exercise and denied any other changes, including fever, chills, nausea and vomiting.

His past medical history was significant for two decades of heavy alcohol consumption (approximately 325 mL whiskey daily), which led to alcoholic cirrhosis, portal hypertension with gastric and esophageal varices, ascites, and a left pleural effusion at the age of 50. The patient had stopped alcohol consumption 18 months prior to the current presentation. He also had a history of diabetes and hyperlipidemia since age 45; gastritis with *H. pylori *infection since age 50; and autoimmune hemolytic anemia since age 51. His oral home medications were Captopril 50 mg daily, Folic acid 1 mg daily, Furosemide 20 mg daily, Glipizide 5 mg daily, Prednisone 80 mg daily, and Spironolactone 50 mg daily.

The patient had no drug allergies and was married, with two adult children. He was employed as an electrician. The patient did not smoke tobacco and had no history of illicit drug use.

His past family history was significant for his mother having been diagnosed with diabetes.

At admission, the patient was alert and in no acute distress, with a pulse of 80/min, blood pressure 104/62 mmHg, respiratory rate 18/min, temperature 99 degrees Fahrenheit, and 100% oxygen saturation on room air. Significant findings on physical exam were slight conjunctival pallor and scleral icterus; a distended abdomen which was minimally tender to palpation and with full, shifting dullness to percussion; and 1+ bilateral lower extremity pitting edema.

**Figure 1:**
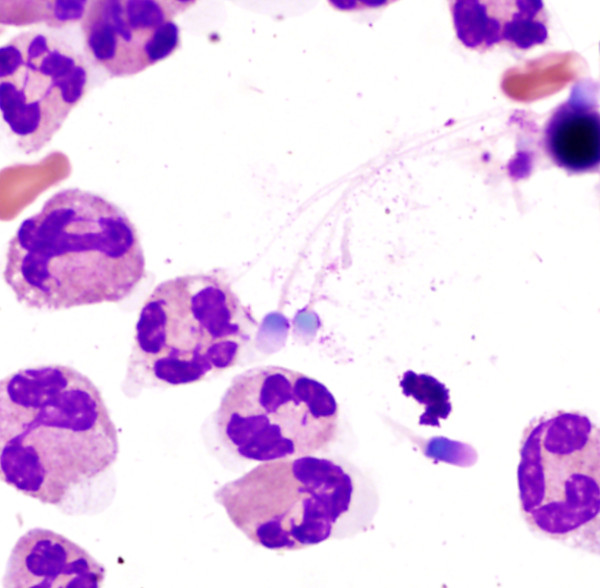
**Peritoneal fluid (Wright-Giemsa stain; 1,000× magnification)**. There are several spermatozoa in a background of frequent neutrophils and occasional red blood cells.

**Figure 2:**
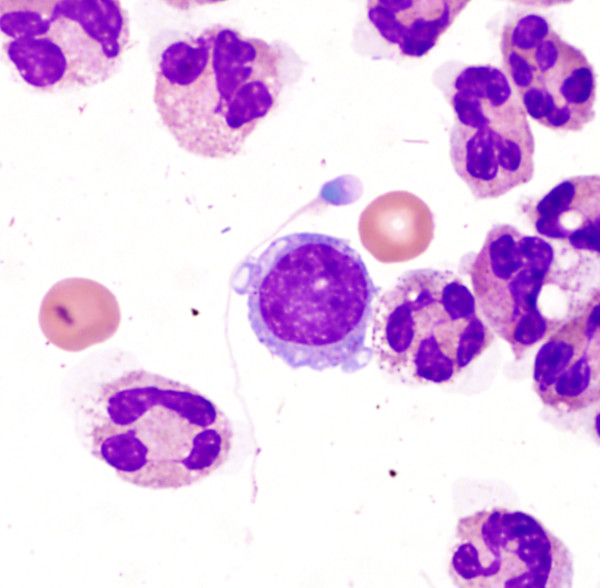
**Peritoneal fluid (Wright-Giemsa stain; 1,000× magnification)**. There is a spermatozoon in a background of frequent neutrophils, rare mononuclear cells and occasional red blood cells.

Diagnostic paracentesis was performed in the emergency room and demonstrated an increased white blood cell count (12,500/μL, 95% neutrophils) and occasional sperm in the peritoneal fluid (Figure 1). Treatment for spontaneous bacterial peritonitis was initiated with intravenous Rocephin. Two days later, a subsequent paracentesis demonstrated a lower white blood cell count (5,500/μL, 51% neutrophils) and persistent rare sperm in the peritoneal fluid (Figure 2). A Urology consult was initiated to further investigate the finding of sperm in the ascitic fluid. The patient reported no history of trauma or instrumentation to the bladder or urethra, no history of a vasectomy, and no history of congenital urogenital defects. The possibility of a bladder rupture with retrograde ejaculation was ruled out by a cystogram that showed no signs of rupture. Furthermore, the fluid creatinine was similar to the serum creatinine and the patient was without urinary symptoms, thus ruling against a urinary ascites. It was concluded that the abdominal tap was either contaminated or the vas deferens had been incidentally injured during the paracentesis procedure. At that point, it was felt that there were no increased risks that would affect his immediate health and no additional workup was recommended. The patient was eventually transitioned to oral Ciprofloxacin (50 mg p.o., b.i.d for 7 days) and discharged in good condition, three days after admission. Throughout his hospital course, the patient remained afebrile and asymptomatic. He had subsequent episodes of spontaneous bacterial peritonitis in the months following his initial presentation; however, no sperm were identified in the ascitic fluid in any of those instances.

## Discussion

**Figure 3:**
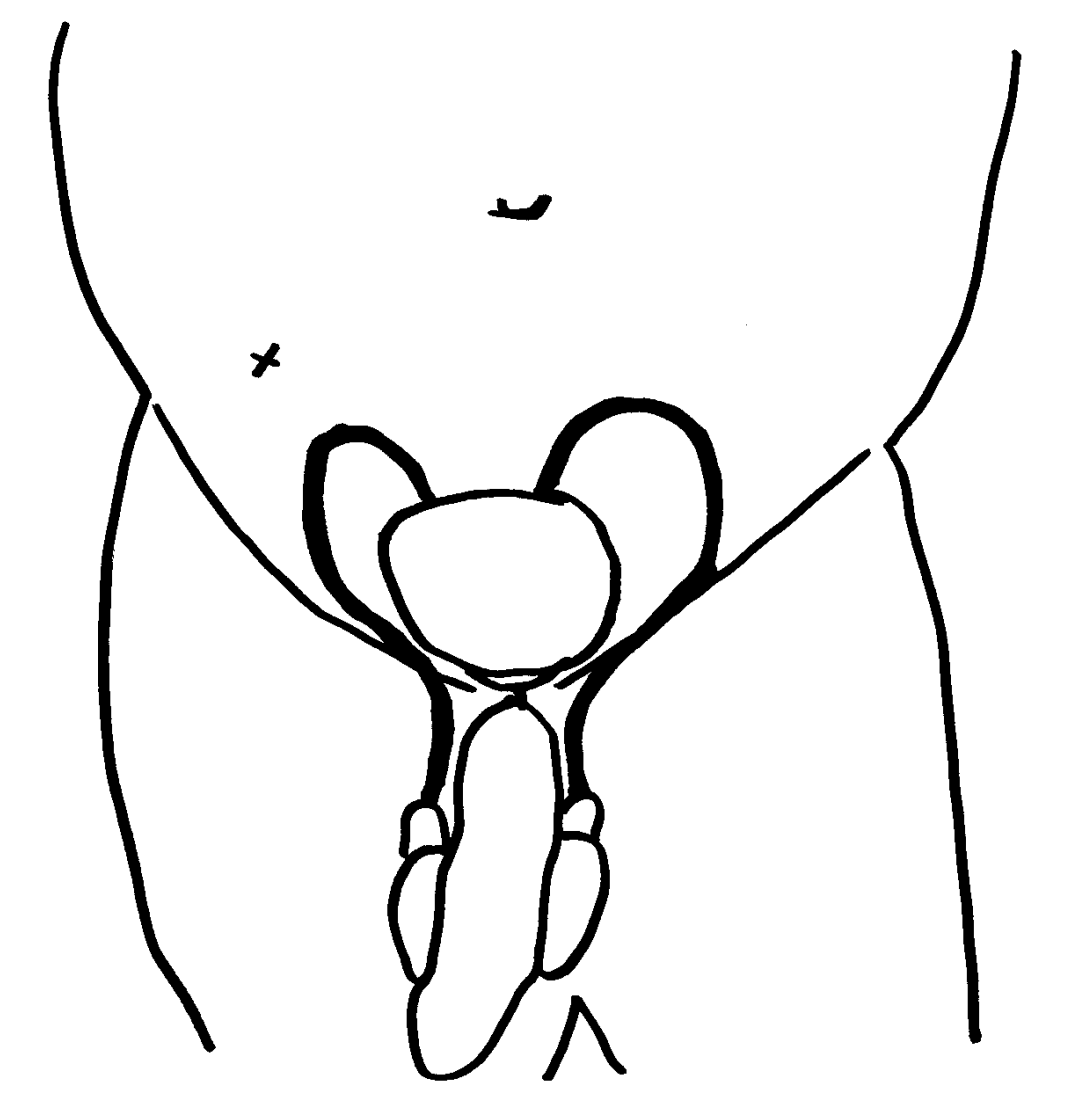
**Schematic representation of the course of the vas deferens, from the epididymis to the seminal vesicle, situated posterior to the urinary bladder**. The approximate position of the paracentesis site is marked with an (x).

The presence of sperm in the peritoneal fluid from a man is very unusual and may be explained by several scenarios. One possibility is that of specimen contamination during specimen collection and processing. A potential source of contamination in the laboratory is a cell counting chamber that was previously used for a sperm count from a semen specimen and then was insufficiently cleaned and used for a different body fluid cell count. In the present case, the possibility of specimen contamination is unlikely, since sperm were identified in two separate collections and the morphology slides were always made from the original container, without intervening pour-over. Another potential cause is that of a bladder defect with retrograde ejaculation, thus allowing sperm to enter the abdominal cavity. This possibility was ruled out by a normal cystogram. Spontaneous recanalization after an intraperitoneal vasectomy is a theoretical risk for sperm being delivered in the peritoneal cavity, but the patient did not have a history of vasectomy. Finally, the vas deferens may have been damaged during paracentesis. This latter explanation was felt to be the most likely event leading to the presence of sperm in the ascitic fluid, although there was no definite evidence to confirm this occurrence. The fluid was obtained through the right lower quadrant, with the peritoneal needle entry at the level of the anterior axillary line intersecting a line connecting the umbilicus with the anterior iliac crest. The needle tract was perpendicular to the skin and it may have inadvertently damaged the vas deferens (Figure 3).

There are anecdotal reports of women becoming pregnant via semen entering the abdominal cavity, secondary to various injuries. One case of so-called "oral conception" describes a patient with aplastic vagina who became pregnant through semen migrating in the peritoneal space following a knife stab wound in the stomach, shortly after she had engaged in oral intercourse [1]. A similar situation was reported during the American Civil War, when a bullet injured the testis of a solider and then became lodged in the abdomen of a young nurse who provided care to the injured nearby. The woman became pregnant and delivered an infant that was later operated on and found to have a bullet lodged in his scrotum. Of note, the woman had an intact hymen and did not report prior sexual intercourse [2].

## Conclusion

To our knowledge, this is the first report of sperm in peritoneal fluid in a male patient. From a patient care perspective, the recognition of this finding was important, since it suggested the presence of additional co-morbidities, which required specific tests in order to be ruled out. It also emphasized that good laboratory practices are paramount in ensuring that unusual findings are not due to specimen contamination in the preanalytical and analytical stage.

## Competing interests

The authors declare that they have no competing interests.

## Authors' contributions

HO was involved in the case management, drafted the manuscript and made the final corrections before submission. AH and SHK were involved in the case management, reviewed the draft manuscript and suggested revisions. All authors read and approved the final manuscript."

## Consent

Written informed consent was obtained from the patient for publication of this case report and accompanying images. A copy of the written consent is available for review by the Editor-in-Chief of this journal.
